# Small world network strategies for studying protein structures and binding

**DOI:** 10.5936/csbj.201302006

**Published:** 2013-03-01

**Authors:** Neil R. Taylor

**Affiliations:** a Desert Scientific Software Pty Ltd, Level 5 Nexus Building, Norwest Business Park, 4 Columbia Court, Norwest, NSW, 2153, Australia

**Keywords:** Small world network, protein structure, protein-ligand binding, protein-protein binding

## Abstract

Small world network concepts provide many new opportunities to investigate the complex three dimensional structures of protein molecules. This mini-review explores the published literature on using small-world network approaches to study protein structure, with emphasis on the different combinations of descriptors that have been tested, on studies involving ligand binding in protein-ligand complexes, and on protein-protein complexes. The benefits and success of small world network approaches, which change the focus from specific interactions to the local environment, even to non-local phenomenon, are described. The purpose is to show the different ways that small world network concepts have been used for building new computational models for studying protein structure and function, and for extending and improving existing modelling approaches.

## Introduction

A fundamental tenet in the study of chemistry is the direct relationship between structure, and chemical and physical properties. In the field of molecular biology, this relationship becomes structure and function [1]. For proteins, structure plays a key role in catalysis, messaging, activation, and disease states [1, 2]. In protein structures, what is particularly interesting is that both rigidity and flexibility are critical properties, often both being needed to fulfil functional requirements [2]. Proteins are synthesised as linear polypeptides that fold into compact three dimensional conformations consisting of a large number of inter-residue close-contacts. They bind with other folded proteins and/or small molecules, often adapting to the shape and electrostatic requirements of these complexes. It is the intrinsic complexity of the three dimensional conformations of proteins, and the important role that proteins play in living organisms, that lead many scientists to study them.

**Figure 1:**
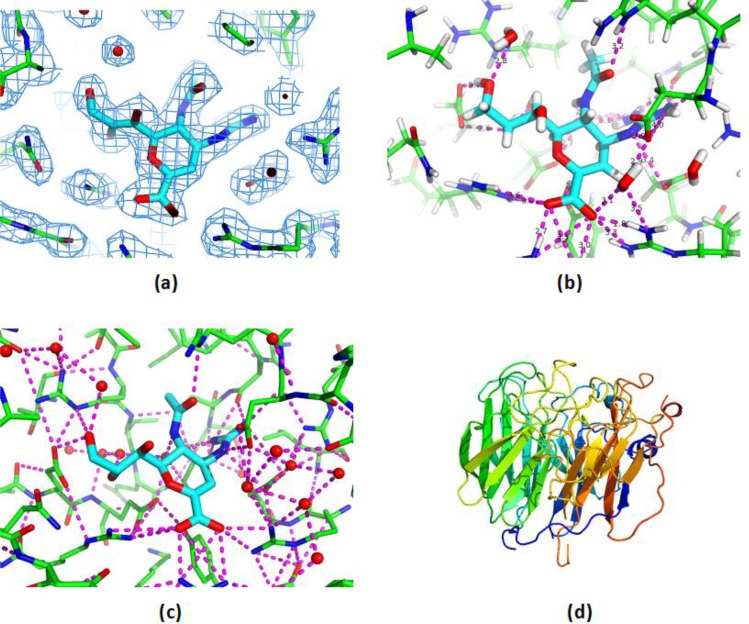
Different representations of 3D protein structure provide different types of understanding. (a) The electron density map for ligand and surrounding binding site residues shows a good quality, rigid model. (b) Typical computational chemistry view, showing that protein-ligand binding involves many short hydrogen bonds. (c) A network view of all favourable polar interactions in the binding site region shows how the protein-ligand hydrogen bonds are parts of highly connected local environments, which also involve numerous bound water molecules. (d) Secondary structure view, suited for structural bioinformatics analysis. Protein is Neuraminidase in complex with Zanamivir. Images generated using PyMol (www.pymol.org), PDB Ids 2cml and 1nnc.

In textbooks, protein structures are described at their different levels of complexity: primary, secondary, tertiary, and quaternary structure [1, 2]. In this article, the focus is on details of three dimensional (3D) structure. In practice, the way that scientists understand and work with 3D structures is closely determined by how they are represented in a computer. Figure 1 illustrates some examples of different representations of 3D protein structure. For protein crystallographers, for example, a protein structure is the atomic model that best represents observed electron density intensities. Atom level details are typically viewed and explored, overlayed with electron density maps. The quality of the electron density measurements and the fit to the atomic model, particularly for key atoms and residues of interest, provide the critical information needed to correctly interpret the data [3].

For computational chemists working in pharmaceutical research, the typical approach is to use a more detailed all atom representation of just the binding site region; usually including hydrogen atoms directed toward their hydrogen bonding partners. An in-depth analysis of the fine-details of specific non-covalent protein-ligand interactions, of local molecular surface shape and electrostatic profile, the role of specific water molecules, etc, provides a vast array of new information for understanding ligand binding [4].

For scientists working in structural bioinformatics, the typical view is often a less detailed schematic representation of 3D structure, highlighting the alpha helices, beta sheets, and loops. Despite the lower detail, this level of analysis provides the best insight into overall topology and stability. Furthermore, comparing and contrasting schematic representations helps with the elucidation of function and functional relationships; leads to powerful classification schemes; and provides insights into evolutionary relationships [5].

This article describes another approach for representing and investigating 3D protein structures, one that has grown rapidly over the last ten years, both in use and scope: the network approach. More specifically, it explores methods that model protein structure as a network of nodes (typically aminoacid residues) and edges (close-contacts between residues) and use a small world network analysis to account for key aspects of structure and function. This type of analysis changes the focus from individual residues and individual interactions to local connectedness and the connectedness of the whole protein. The change allows non-additivity effects and non-local effects to be incorporated into modelling. An example network view for a protein is shown in Figure 1c.

The aim of this paper is to review the different ways that small world network concepts have been used as the foundation for building new computational models to study protein structure and function, and to extend and improve existing modelling approaches. The focus is on cheminformatics and bioinformatics, describing when and how network descriptors have been combined with standard properties to account for the contributions of non-local and global effects. A brief introduction to networks and different network types, and the main descriptors used to explore them, is provided. A short historical perspective on small world networks is also included. Since the focus is on molecular structure, in the main part of this review the literature is divided according to whether the research is on single proteins, protein-ligand binding, or protein-protein binding.

### Introduction to Networks

When a system is defined as a network, one is describing it at a highly abstract level, reducing it to a collection of simple nodes joined to one another by simple edges. It should be mentioned that the terms network and graph can be used inter-changeably, as can node/vertex, and also edge/connection/wiring. Network studies are less interested in the attributes of individual nodes and edges, and more interested in the properties arising from local and global topologies that are the result of the connectedness of the nodes, see for example Bollobas [6]. Typically, edges do not have a directional component, nor do they have different strengths (networks of these types are described as unweighted, undirectional graphs). Two important properties when describing any network are node degree, the count of the number of edges a node has, and shortest path length -the count of the minimum number of edges needed to be traversed to get from one node to another. Key information can be obtained from both the averages and distribution functions of these values over an entire network [6].

The main network types encountered in the literature are regular, random, small-world, and scale-free. A regular network is a regular array of nodes and edges, with high local symmetry -all nodes having identical connectivity -in other words, a regular lattice or mesh. An example of this type of network, well known to all scientists, is the diamond lattice, the arrangement of atoms and covalent bonds in diamond. A random network is one in which edges occur randomly, that is, the rules governing attachment are entirely random (not based on node proximity, for instance). In a random network, most nodes have approximately the same number of edges, and node degree has a Poisson distribution [6]. A small world network typically arises when one very simple rule governs attachment, that rule being “popularity is attractive“ [7, 8]. That is, a new node in the network is more likely to form an edge with a node that already has a higher than average number of attachments, or with another node that has a short path length to such a node. A scale-free network can be thought of as an extreme case of small world behaviour, having a degree distribution that follows a power-law distribution. In a scale free network it can be possible to find nodes with degree five to ten log units higher than the average degree [8] -such nodes can never possibly arise in random networks. Hub is the name given to the few nodes with the highest number of links.

**Figure 2:**
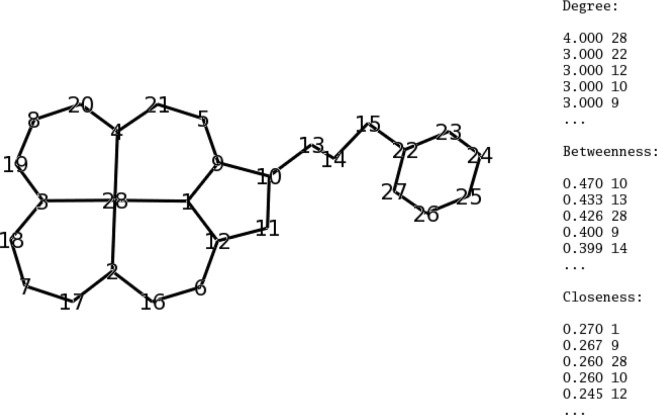
Simple chemical graph showing the three most widely used network descriptors for nodes, with the top five values for each. Node 28 has the highest degree, that is, the most connections (and is therefore a hub). Node 10 has the highest betweenness, which is a function of the fraction of shortest paths through a node (removing this node creates the two largest disconnected fragments). Node 1 has the highest closeness, that is, the inverse of the average of the shortest paths to all other nodes. This particular graph has a low clustering coefficient - no pair of connected nodes is connected to the same third node (no triangles). The characteristic path length is low (near 5) though this is not particularly meaningful as it is only a very small network.

Global network descriptors enable different types of networks to be distinguished from one another. Within a graph, local descriptors can be used to distinguish the different roles played by different nodes, based just on the connections. The most commonly used local network descriptors for nodes are explained in Figure 2. The two most commonly used global network descriptors are: characteristic path length, L, the average of the shortest path lengths between all pairs of nodes (small for random graphs); and clustering coefficient, C, the average over all nodes of the fraction of the number of connected pairs of neighbours for each node (large for regular networks). Watts and Strogatz [9] showed that the different network types have different magnitudes and ratios of L and C: regular networks have large L and large C; random networks have small L and small C; and small world networks have small L and large C.

A number of introductory books have been written on the subject of small world networks [10, 11]. For readers interested in a more detailed coverage of the underlying physics and mathematical aspects, Dorogovtsev et al. [8] is recommended. A comprehensive survey of the scientific literature is available in Boccaletti et al. [12], with nearly 900 references; within the field of medicines research, including protein structure networks as discussed herein, see Csermely et al. [13] which has more than 1100 cited references. Surveys, introducing more of the available literature on protein structure networks, include Boede et al. [14] and also Krishnan et al. [15].

A wide range of powerful and versatile software tools are available for researchers interested in using network approaches in their work, such as Cytoscape [www.cytoscape.org], NetMiner [www.netminer.com], Networkx [networkx.lanl.gov], JUNG [jung.sourceforge.net]. See Csermely et al. [13] for many more examples. In the field of protein structure networks, on-line research tools are also available, including RING [protein.cribi.unipd.it/ring], RINalyzer[www.rinalyzer.de], and Scorpion [www.desertsci.com].

## Historical perspective

For many years, the study of networks focused on the properties of random graphs with normal or Poisson distributions of connections between nodes [16–17]. The focus changed in the late 1990s in parallel with the incredibly rapid growth and development of the internet and the world wide web. Subsequently, the world wide web was observed to have: (1) an unexpectedly low average shortest path length between any pair of nodes, and (2) a fat-tailed degree distribution, that is, the number of connections for some nodes is many orders of magnitude higher than the average number [7]. They termed these small world networks, and numerous studies have found them throughout nature [7, 18–22]. Not only have they been identified in many different realms in the natural world, but also in man-made systems including communications systems, financial systems, scientific citations, and throughout numerous human social organisations [8]. The reason why small world networks are so frequently observed is believed to be due to their stability -stability here meaning maintaining integrity and minimising the possibility of failure. This stability is believed to arise from optimised communication pathways within these networks, or in other words, from short path lengths between all parts of the network. Since 2002, small world network concepts have been incorporated more and more into the fields of chemistry and structural biology [13]. The thinking behind using the network approach in protein structure analysis is that it allows for contributions from non-local effects to be included into a model.

To clarify, there are two completely different fields of research involving proteins and networks. The subject of this paper is the field of protein conformation. The other field, which has been more widely investigated and reported, involves communication pathways between whole proteins, and commonly referred to as protein networks, protein contact networks, or protein-protein interaction networks (see Csermely et. al. [13] for an overview). That field lies more within biology, specifically systems biology, than within chemistry.

## Individual protein domains

Many articles have been published describing protein domains using small world network approaches, based on the network of proximal aminoacid residues [23–45]. Network analyses of aminoacid graphs have been used to tackle many different aspects of topology, stability, folding, and communication pathways. Analysis typically involves a comparison of the calculated characteristic path length and clustering coefficient against a [comparable] random network. Articles consistently report that protein domains have small world network characteristics, or more precisely, have topologies that lie somewhere in the middle between the two extremes of random networks and scale free networks. The network of aminoacid sidechains cannot be fully scale free due to two important boundary conditions. The first is the limit of packing multiple residues in the coordination sphere of a central residue, also known as the excluded volume affect; the second involves the numerous constraints imposed by the protein backbone, for example, that residue i is covalently constrained by residues i-1 and i + 1. Despite these constraints, evidence that small world network effects contribute to the structural integrity of protein structures is highly persuasive. Mention is sometimes made of the role of evolution: that proteins are the product of evolutionary optimisation processes, and that topologies with small world network characteristics have been selected due to advantages provided for overall stability, robustness, and protection against failure of function due to mutations [27]. The point is often made that modelling a protein structure as network means modelling as a communication system, which provides many opportunities for new insights into structure and function [34, 42, 44].

Table 1 lists a subset of articles, demonstrating the broad scope of the problems studied, and methods used. In the earlier articles on residue networks for protein domains (and complexes), graphs were built using C alphas as the nodes (or C betas) and an edge defined when two nodes were within a threshold cutoff, typically in the range 7.0 to 9.0 Angs. It is now more common to define edges using a higher level of atomic detail - when a pair of heavy atoms is within a threshold cutoff, typically in the range 4.0 to 5.0 Angs. Although this method is more consistent with the energetics of atom-atom close-contacts, it has the drawback of not allowing for through-water interactions between residues, or through-metal interactions. There are many different descriptors that can be computed for any network, however, on their own, these descriptors do not provide such interesting predictions for proteins. It is when they are combined with other structural and sequence properties, or added into existing models, that they do become really useful. The purpose of listing the different methods in Table 1 is to provide a comprehensive overview of the different cheminformatics and bioinformatics methods that have been explored to date, so that these may provide ideas for further research.

**Table 1:** Overview of publications using network methods to model the three dimensional conformations of protein domains. The summaries focus on the different cheminformatics and bioinformatics methods employed, and the properties investigated.

Publication	Topic of Inquiry	Methods Used
Dokholyan et al. 2002 [24]	Studied folding by creating networks based on conformations generated from Monte Carlo simulations	Showed that post-transition state protein conformations are more small-world like, that is, globally tighter, than pre-transition state conformations. Characteristic path length was found to be the best performing global descriptor examined
Vendruscolo et al. 2002 [25]	Studied folding by creating a weighted graph from transition state ensembles of protein structures generated by Monte Carlo sampling	Edge weights obtained by dividing the number of structures with a specific edge by the total number of structures in the ensemble. Betweenness values were highest for key residues involved in folding
Greene et al. 2003 [26]	Studied protein conformation	Used a network with weighting factors derived from the presence of multiple links between residues
Brinda et al. 2005 [32]	Studied protein structure and stability	Used edge weighting based on the number of close contacts between a pair of residues divided by normalisation factors (which take into account residue size and the propensity to make a large number of contacts) for each residue, and allowing for varying interaction strengths
Paszkiewicz et al. 2006 [33]	Used network methods to identify a predictor of suitable regions of circular permutation	Closeness was shown to useful descriptor; exploration of relative side chain area and sequence conservation measures also undertaken
del Sol et al. 2006 [34]	Study of communication aspects of the aminoacid residue network	Used a characteristic path length metric, corresponding to the change in path length resulting from removal of a node
Muppirala et al. 2006 [35]	Aim was to distinguish correctly folded from incorrectly folded proteins	Used more stringent distance constraints for hydrogen bonding (and also angle cutoffs), and similarly for hydrophobic and ionic contacts, and disulphide bonds, and conserved hydrophobic aminoacids were mapped onto the network to examine their roles
Aftabuddin et al. 2007 [36]	Protein structure analysis	Separated amino acid sidechains into different types, hydrophobic (F, M, W, I, V, L, P, A), hydrophilic (N, C, Q, G, S, T, Y), and charged (R, D, E, H, K), and the weighted (according to the number of close-contacts between a pair of residues) and unweighted networks separately analysed
Gaci et al. 2008 [41]	Examined subgraphs involving residues participating in secondary structure elements, alpha helices and beta sheets	Explored distribution of characteristic path length and clustering coefficient as a function protein size
Li et al. 2008 [42]	Examined protein folding and communication between residues	Found that key residues in the folding process -found to be global centrals rather than local centrals -could be identified using solvent accessibility and network terms together
Milenkovic et al. 2009 [43]	Sought to determine the best null model for discriminating network motifs, including secondary structure motifs, in aminoacid graphs	Examined different choices for nodes -just using side chain atoms, just using backbone atoms, using all residue atoms -and different wiring models explored, including 3D geometric, random, scale free, and stickiness-index based networks
Vijayabaskar et al. 2010 [44]	Investigated protein structure integrity and communication pathways using energy weighted networks	Protein energy networks (PENs) were created with edges weighted according to calculated interaction energies, obtained from sampling structures from a molecular dynamics simulation, and fluctuations from the mean
Petersen et al. 2012 [45]	Protein structure analysis, seeking patterns in the packing of amino acid pairs	Created an eight dimensional descriptor space encompassing residue proximity, solvent accessibility, sequence distance, secondary structure, and sequence length, and uncovered a scale free organization in aminoacid pair interactions

## Active site analysis and protein-ligand binding

The small world network approach has been particularly successful in the prediction of binding site residues [27–29, 46–50], for example, using closeness centrality with solvent accessibility scores [27]; combining closeness centrality and phylogenetics [29]. Betweenness centrality, when averaged over a patch of residues, was found to improve the predictive power of a model for identifying residues involved in binding RNA [48]. Heme-binding residue prediction [49] was shown to work well using standardised network descriptors in combination with a sequence profile descriptor and several additional structural descriptors, including solvent accessibility, measures of local concavity and convexity of the protein surface. Interestingly, the clustering coefficient was found to be lower for residues that bind heme, that is, they are less packed, suggested to be important for allowing for some flexibility in the binding site. Network descriptors have also been incorporated into a method for predicting DNA-binding residues [50], with a weighted average betweenness centrality measure combined with features from evolutionary profiles, interface propensity, and side-chain solvent accessibility. In a network study of DHFR [47], the network model included the nitrogen and oxygen atoms of the cofactor. Changes in closeness values showed that cofactor binding, specifically at the binding site, has a significant affect on the network, and that in the complex, most network interactions involved the cofactor. Ligand binding sites are unique regions on the surfaces of proteins, and the ability of network approaches to successfully identify these regions strongly hints at the importance of including network effects into modelling.

A small world network approach has also been used to build a quantitative model for predicting ligand binding affinity [51]. In this work, the small-world network architecture of protein structure was assumed, and a novel set of localised network descriptors developed. A reduced graph definition of protein structure was used, with separate nodes for each sidechain and mainchain group (including C alpha), and included HET groups, metal atoms, and tightly bound water molecules. Network edges were created from all covalent and favourable non-covalent interactions between atoms, identified using a comprehensive classification scheme. Network descriptors were constructed in such a way as to maximise potential small-world network influences, and incorporated all short paths, rather than just shortest paths, as in most previous studies. The network model served to increase the affinity contributions of non-covalent interactions when the local environment was more highly connected. In this work, global descriptors were found not to be generally applicable as they were overly sensitive to individual close-contacts. In some cases ligand binding affinity was found to track closely with ligand molecular weight, and additional descriptors did not significantly improve predictions. In other cases, tight binding clearly involved additional factors, and the incorporation of non-local affects [via the network model] helped to account for these.

## Protein-protein binding

Models based on residue interaction networks have been used in a number of studies of protein-protein complexes, see for example [52–54]. In an exploration of protein-protein interaction sites, conserved residues with high betweenness scores, buried upon dimerisation, correlated well with experimentally determined hotspot residues [52]. At the same time, network descriptors for uncomplexed monomers were found not to be good predictors for the protein-protein interface - the majority of high-betweenness values at the interface are created with dimerisation.

Several small world network approaches have been developed to improve the performance of in silico protein-protein docking [54, 55]. Chang et al. [54] computed network descriptors from two networks, one with hydrophobic residues as nodes (Ile, Leu, Val, Phe, Met, Trp, Cys, Tyr, Pro, Ala) and one with hydrophilic residues as nodes (Gly, Lys, Thr, Ser, Gln, Asn, Glu, Asp, Arg, His). Edges were created for every atom pair within 5.0 Angs of one another (for atoms from two different residues). The two networks themselves were shown to be small-world networks. Clustering coefficient and characteristic path length were computed, then combined with other parameters, which included terms for vdW interactions (attractive and repulsive), solvation, hydrogen bonding, long-range electrostatics (attractive and repulsive), constraining side chains rotamers, and a residue-residue pair probability metric. To account for differences in protein size, network parameters were standardised by calculating their standard deviation from the mean for each protein. Parameterisation was done by maximally separating true docked solutions from decoys. They showed that the addition of the new network terms to the scoring function improved the discrimination capabilities of the method. Interestingly, they observed that correct docking solutions exhibit lower characteristic path lengths than incorrect solutions, suggesting that correct solutions better preserve the characteristic path length of native protein structures than incorrect solutions.

Pons et al. [55] achieved improved results using network descriptors to help score rigid body protein-protein docking solutions. Apo proteins were modelled as networks, with the C alpha atoms of all aminoacids as nodes, and edges when two C alpha atoms were within 8.5 Angs (results mostly unchanged if C beta atoms used instead). Four different network parameters: degree, closeness, betweenness, and clustering coefficient, were investigated to determine their utility in protein-protein docking studies. Best results were obtained when combining calculated closeness descriptors with the default scoring function [56]. The article is also of interest in that a number of diverse influences were explored to determine under which conditions the network descriptors work best, including the type of complexes, flexibility, complex size, and protein shape.

## Summary and Outlook

The published literature on using network models to study protein structure consistently shows that the structures exhibit small world characteristics. In addition to the experimental support provided by analysis of characteristic path length and clustering coefficient, further verification is provided by the success of incorporating network descriptors into existing models and observing better quality predictions. The small world network approach is attractive from the conceptual perspective, providing better understanding of the stability of protein topology, both in terms of overall structural integrity and in terms of robustness against failure of function due to mutations. The network approach is also attractive from the modelling perspective, enabling non-local and global affects to be incorporated into computational methods.

In this review it has been shown how small world network approaches have successfully contributed to a range of studies involving protein structure and function. The network approach has been shown to help investigation of global phenomena, such as protein folding, and local phenomena, such as ligand binding and protein-protein docking. The network approach itself is highly abstract and so really useful results cannot be achieved by network descriptors alone, that is, they need to be combined with other factors: for example, with physico-chemical properties and/or sequence conservation measures; or added onto existing models. A list of different methods using network descriptors has been included to provide new researchers in the field with a better understanding of what has been done and possible directions for future research.

Following recent reported success using descriptors derived from network approaches, particularly in protein-ligand binding and protein-protein docking, one could expect to see more widespread use of network based methods, particularly to augment existing models. As algorithms improve and computer hardware becomes more powerful, one would expect to see more investigations using weighted graphs. Furthermore, as experience grows using network descriptors in work with proteins, one would hope to see a wider range of descriptors explored, not limited to the descriptor types used in other fields. Sidechain inter-connectivity in proteins has been fine-tuned by constraints imposed by nature, of packing residues that are covalently linked to one another, and so the network descriptors that best fit this field are probably unlike the descriptors suited to other areas of study. The small world network concept seems well suited for investigating the role of water molecules in protein stability and binding events (which is a current topic of high interest), and for studying protein flexibility, which also has important functional implications. Future research will provide a better understanding of the conditions under which network approaches work best, and when they should be included into research programs.
